# Design and level of evidence of studies published in two Brazilian medical journals recently indexed in the ISI Web of Science database

**DOI:** 10.1590/S1516-31802010000400005

**Published:** 2010-07-01

**Authors:** Maria Regina Torloni, Rachel Riera

**Affiliations:** I MD, PhD. Gynecologist and obstetrician in the Department of Obstetrics, and research assistant in the Brazilian Cochrane Center, Universidade Federal de São Paulo — Escola Paulista de Medicina (Unifesp-EPM), São Paulo, Brazil.; II MD, MSc. Research assistant in the Brazilian Cochrane Center, and physician in the Discipline of Emergency Medicine and Evidence-Based Medicine, Universidade Federal de São Paulo — Escola Paulista de Medicina (Unifesp-EPM), São Paulo, Brazil.

**Keywords:** Journal impact factor, Periodicals as topic, Journal article [Publication type], Research design, Review [Publication type], Fator de impacto de revistas, Publicações periódicas como assunto, Artigo de revista, Projetos de pesquisa, Revisão

## Abstract

**CONTEXT AND OBJECTIVES:**

The level of evidence and methodological quality of articles published in medical journals are important aids for clinicians in decision-making and also affect journals’ impact factor. Although systematic reviews (SR) are considered to represent the highest level of evidence, their methodological quality is not homogeneous and they need to be as carefully assessed as other types of study. This study aimed to assess the design and level of evidence of articles published in 2007, in two recently indexed Brazilian journals (Clinics and Revista da Associação Médica Brasileira), and to evaluate the methodological quality of the SRs.

**DESIGN AND SETTING:**

Descriptive study developed in the Brazilian Cochrane Center, Universidade Federal de São Paulo.

**METHODS:**

All 289 published articles were classified according to types of study design and level of evidence. The SRs were critically appraised by two evaluators using the AMSTAR tool.

**RESULTS:**

The most frequent design types were cross-sectional studies (39.9%), case reports (15.8%), experimental studies (10.8%) and narrative reviews (7.4%). According to the Oxford criteria, 25.6% of the articles were classified as level 4 or 5 evidence, while 2.8% were level 1. SRs represented only 2% of the published articles and their methodological quality scores were low.

**CONCLUSIONS:**

The main design types among the published papers were observational and experimental studies and narrative reviews. SRs accounted for a small proportion of the articles and had low methodological scores. Brazilian medical journals need to encourage publication of greater numbers of clinically relevant papers of high methodological quality.

## RESUMO

**CONTEXTO E OBJETIVOS:**

O nível de evidência e a qualidade metodológica dos artigos publicados em revistas médicas são importantes para auxiliar os clínicos a tomarem decisões e também afetam o fator de impacto dos periódicos. Apesar de as revisões sistemáticas (RS) serem consideradas o maior nível de evidência, sua qualidade metodológica não é homogênea e elas precisam ser avaliadas tão criteriosamente como outros tipos de estudos. O objetivo deste estudo foi avaliar os desenhos e nível de evidência dos artigos publicados em 2007 em duas revistas brasileiras recentemente indexadas (Clinics e Revista da Associação Médica Brasileira) e avaliar a qualidade metodológica das RS.

**TIPO DE ESTUDO E LOCAL:**

Estudo descritivo desenvolvido no Centro Cochrane do Brasil, Universidade Federal de São Paulo.

**MÉTODOS:**

Todos os 289 artigos publicados foram classificados quanto ao tipo de desenho e nível de evidência. As RS foram analisadas criticamente por dois revisores usando o instrumento AMSTAR.

**RESULTADOS:**

Os tipos de desenhos mais frequentes foram os transversais (39,9%), relatos de casos (15,8%), estudos experimentais (10,8%) e revisões narrativas (7,4%). Segundo os critérios de Oxford, 25,6% dos artigos eram de nível 4 ou 5 e 2,8% de nível 1. As RS representaram apenas 2% do total dos artigos publicados e tiveram notas de qualidade metodológica baixas.

**CONCLUSÕES:**

Os principais tipos de desenhos dos estudos publicados foram observacionais, experimentais e revisões narrativas. As RS representaram uma pequena parcela dos artigos e tiveram baixa pontuação metodológica. Revistas médicas brasileiras devem incentivar a publicação de mais artigos clinicamente relevantes com alta qualidade metodológica.

## INTRODUCTION

Evidence-based medicine consists of conscientious, explicit and judicious use of the current best evidence in making decisions about care for individual patients.^1^ Busy clinicians, especially in developing countries, frequently rely on medical journals to help them find reliable evidence to answer questions that come up in their daily practices. In this context, medical journals can play an important educational role by offering high-level evidence to help and support these clinicians in their decision-making processes.

Systematic reviews (SRs) are currently considered to be the highest level of evidence in the hierarchy of studies.^2^ SRs involve an exhaustive review of the literature addressing a clearly defined question, using systematic, transparent and explicit methodology to identify, select and critically evaluate all the relevant studies. After relevant data from the primary studies available have been collected, extracted and analyzed, a synthesis of the findings is then produced in a clear and objective manner. Conducting a SR is a complex task and flaws are possible in this process, thus leading to variations in the quality of published SRs. Therefore, readers and users of SRs should take a critical viewpoint and look carefully at the methodological quality of the published papers available.

From an editorial perspective, the methodological quality and level of evidence of the articles published in a journal are important determinants of how often an article is cited, and they consequently affect the impact factor (IF) of that journal. Eight Brazilian medical journals were recently included in the Journal Citation Reports (JCR) database^3^ and will have their impact factor available from 2010 onwards. Two recently published articles^4,5^ have assessed the relevant methodological aspects of articles published in 2007 in four of these journals (Acta Ortopédica Brasileira, Revista Brasileira de Medicina do Esporte, Arquivos Brasileiros de Cardiologia and Revista Brasileira de Cirurgia Cardiovascular). As a continuation of this series, the present article will analyze the articles published in two additional journals (Clinics and Revista da Associação Médica Brasileira) during the same year, looking specifically at the number and quality of published systematic reviews.

## OBJECTIVES

This review aimed to analyze the types of articles published in 2007 in two recently indexed Brazilian medical journals. Specifically, we sought 1) to assess the distribution of articles according to type of study design and level of evidence, and 2) to evaluate the methodological quality of published systematic reviews.

## METHODS

Through a manual search, the two authors retrieved all articles published in 2007 in Clinics and Revista da Associação Médica Brasileira (RAMB). All manuscripts were read and classified according to their study design into one of eight main categories: 1) Primary clinical studies: case reports and case series, case-control studies, cohort studies, cross-sectional studies, trials (randomized, quasi-randomized and non-randomized) and diagnostic accuracy studies; 2) Primary experimental studies on animal models or corpses; 3) Integrative studies on the literature: narrative reviews and systematic reviews; 4) Technical notes describing surgical procedures or anatomical findings; 5) Development, translation and validation of scales, clinical measurements and questionnaires; 6) Clinical guidelines; 7) Continuing medical education quizzes; and 8) Others: editorials, commentaries (on other published studies), opinions, reflections on practice and attitudes and letters to the editor. Each article was classified after reading the title, abstract (when available) and methods sections.

Each article was classified according to the system of the Oxford Centre for Evidence-based Medicine Levels of Evidence.^6^ This classification system ranks the validity of the evidence into a hierarchy, such that level 1 is the highest level and level 5 is the lowest. Articles categorized in groups 4-8 (non-evidence literature) were excluded from this analysis.

To achieve the second objective, the full texts of all the systematic reviews published in these two journals in 2007 were retrieved, read and evaluated by two independent raters (the authors) using the AMSTAR tool (a measurement tool to assess systematic reviews), which consists of 11 items that are rated as 0 or 1.^7^ This tool has good face and content validity for measuring the methodological quality of systematic reviews.^8^ The AMSTAR scores of each rater were compared and differences were discussed until a consensus was reached.

## RESULTS

In 2007, Clinics and RAMB each published five issues, containing 127 articles and 162 articles, respectively. Thus, a total of 289 articles were evaluated. Table 1 presents the distribution and relative frequency of each type of article published in the two journals. Cross-sectional studies and case reports were the most frequent design type in both journals, representing over 50% of all the clinical articles published during that year. Out of the 289 articles published in 2007, four were randomized (or quasi-randomized) trials^9-12^ and four were systematic reviews,^13-16^ thus representing less than 2% of the total number of published papers in both of these journals. After exclusion of six clinical guidelines^17-22^ and 80 non-evidence articles (technical notes, clinical measurement validation studies, continuing medical education, quizzes and others) the distribution of the remaining 203 articles is presented in Table 2. According to the Oxford criteria, 25.6% of the articles were classified as level 4 or 5 evidence, while 2.8% were level 1.

**Table 1.** Distribution of all articles published in two Brazilian journals in 2007

Type of article	Clinics 2007		RAMB 2007	
	n	%	n	%
**1. Primary clinical articles**	89	70.1	73	45.1
cross-sectional study	29	22.8	52	32.1
case report	26	20.5	6	3.7
prospective cohort study	10	7.9	2	1.2
case series	7	5.5	2	1.2
retrospective cohort study	7	5.5	6	3.7
accuracy diagnostic study	4	3.1	2	1.2
RCT or quasi-RCT	3	2.4	1	0.6
non-randomized trial	2	1.6	2	1.2
case-control study	1	0.8	0	0.0
**2. Experimental studies**	14	11.0	8	4.9
**3. Integrative studies**	8	6.3	11	6.8
narrative review	7	5.5	8	4.9
systematic review	1	0.8	3	1.9
**4. Technical notes** *	1	0.8	1	0.6
**5. Clinical measure validation**	4	3.1	1	0.6
**6. Clinical guidelines**	0	0.0	6	3.7
**7. CME quizzes based on guidelines**	0	0.0	6	3.7
**8. Other**	11	8.7	56	34.6
commentary	0	0.0	19	11.7
editorial	9	7.1	15	9.3
letter to editor	2	1.6	4	2.5
opinion	0	0.0	18	11.1
**Total**	127	100.0	162	100.0

* Study describing surgical technique or anatomical study.

CME = continuing medical education; RCT = randomized controlled trial; RAMB = Revista da Associação Médica Brasileira.

**Table 2.** Study designs of evidence-providing articles published in 2007 in Clinics and Revista da Associação Médica Brasileira (RAMB)

Type of article	Clinics 2007		RAMB 2007		Both journals	
	n	%	n	%	n	%
**Clinical articles**						
cross-sectional study	29	26.1	52	56.5	81	39.9
case report	26	23.4	6	6.5	32	15.8
prospective cohort study	10	9.0	2	2.2	12	5.9
case series	7	6.3	2	2.2	9	4.4
retrospective cohort study	7	6.3	6	6.5	13	6.4
accuracy diagnostic study	4	3.6	2	2.2	6	3.0
RCT or quasi-RCT	3	2.7	1	1.1	4	2.0
non-randomized trial	2	1.8	2	2.2	4	2.0
case-control study	1	0.9	0	0.0	1	0.5
Subtotal	89		73		162	79.8
**Experimental studies**	14	12.6	8	8.7	22	10.8
**Reviews**						
narrative review	7	6.3	8	8.7	15	7.4
systematic review	1	0.9	3	3.3	4	2.0
Subtotal	8		11		19	9.4
**Total**	**111**	**100.0**	**92**	**100.0**	**203**	**100.0**

RCT = randomized controlled trial.

Table 3 presents the AMSTAR scores of the four systematic reviews^13-16^ published during 2007 in the two journals. The overall scores ranged from 1 to 6, with a mean score of 4.0 (standard deviation: 2.2).

**Table 3.** Methodological quality of systematic reviews published in Clinics and Revista da Associação Médica Brasileira (RAMB), 2007

Review	AMSTAR item											
	1	2	3	4	5	6	7	8	9	10	11	Total
Couto et al.^13^	1	0	0	0	0	1	1	1	1	1	0	**6**
Faria et al.^14^	1	0	0	0	0	1	1	1	0	0	0	**4**
Holz et al.^15^	0	0	0	0	0	0	1	0	0	0	0	**1**
Raimondi^16^	1	0	0	1	0	0	1	1	1	0	0	**5**

Scale for item score: 0 = absent; 1 = present.

The AMSTAR (a measurement tool to assess systematic reviews) criteria are as follows: (1) a priori design; (2) duplicate study selection and data extraction; (3) comprehensive literature search; (4) inclusive publication status; (5) included/excluded studies provided; (6) characteristics of included studies provided; (7) quality assessment of studies; (8) study quality used appropriately in formulating conclusions; (9) appropriate methods used to combine studies; (10) publication bias assessed; and (11) conflict of interest stated.

## DISCUSSION

The most frequent study designs of the articles published in Clinics and RAMB in 2007 were cross-sectional studies, case reports, experimental studies and narrative reviews, which together represented about three-quarters of all the clinical papers published. The number of systematic reviews published in these two journals represented only 2% of all the clinical papers published. Their methodological quality, as assessed by AMSTAR, was low.

With rare exceptions, case reports, experimental studies or narrative reviews are insufficient to justify or support healthcare decisions regarding treatment, diagnosis or prevention. However, we found that a relatively large number of such articles were published in the two journals. The journal Clinics contained a large number of experimental studies (n = 14), mostly using rat models, but also one study using a pig model (analyzing respiratory dysfunction in relation to sepsis)^23^ and three experimental studies on the knees of human cadavers.^24-26^ For both journals, the most common clinical questions addressed in the articles related to the prevalence, frequency or associations between variables, which were answered using cross-sectional studies. There were only 25 cohort studies (8.6% of all the articles), and a single case-control study.^27^ The popularity of cross-sectional studies is probably due to the fact that they are methodologically easier to conduct, require less time and cost less than other types of observational studies (cohort and case-control studies) or clinical trials.^28^ These practical considerations are important factors in determining what type of study to conduct, especially in settings with limited resources.

According to the quality assessment tool used, the few systematic reviews published in the two journals were of relatively low quality. To reduce bias and subjectivity, it is recommended that all steps of a systematic review be conducted by two independent investigators. However, one of the published reviews was apparently performed by a single reviewer (the author)^16^ and, although the other three reviews had more than one author,^13-15^ they did not report whether the study selection and data extraction had been done in duplicate. Similarly, none of the four reviews provided all the necessary details on the literature search, such as the names of the electronic databases, the years and key words used, and whether searches were complemented by consulting current contents, reviews, textbooks, specialized registers or experts in the particular field of study, or by reviewing the references in the studies found. Additionally, none of the reviews provided a complete list of the studies included and excluded, nor did they clearly acknowledge potential sources of support, either for the systematic review or for the studies included.

Ideally, all published medical articles should be relevant, have good methodological quality and offer a high level of evidence. Although this may be the sincere aim of the editors of most journals, the reality for the reviewers and editorial teams of medical journals is that they have to struggle through piles of submitted manuscripts every week, trying to sort and select for publication the best possible studies from among a plethora of less-than-perfect texts submitted by hopeful authors. Nevertheless, in keeping with the goal of offering high-level evidence for clinicians, and in order to increase their impact factor, Brazilian medical journals should be encouraged to try to increase the numbers and methodological quality of the systematic reviews published in their issues.

## CONCLUSIONS

In 2007, the most common study designs among the articles published in Clinics and RAMB were cross-sectional studies, case reports, experimental studies and narrative reviews. The numbers and methodological quality of the systematic reviews published in these two journals were low. Publication of greater numbers of clinically relevant and high-quality studies should be encouraged among Brazilian journals.
